# Rotation-based metric on the Riemannian manifold of SPD matrices with applications to source data selection for brain-computer interface transfer learning

**DOI:** 10.3389/fnhum.2026.1824613

**Published:** 2026-05-21

**Authors:** Frida Heskebeck, Bo Bernhardsson, Carolina Bergeling

**Affiliations:** 1 Department of Automatic Control, Lund University, Lund, Sweden; 2 Department of Mathematics and Natural Sciences, Blekinge Institute of Technology, Karlskrona, Sweden

**Keywords:** BCI, pole ratio, Riemann geometry, source data selection, transfer learning

## Abstract

This paper introduces the pole ratio metric and presents a sphere-based view of symmetric positive-definite matrix rotations on the Riemannian manifold of symmetric positive-definite matrices equipped with the affine-invariant Riemannian metric. The pole ratio quantifies whether data from different users lie on this Riemannian manifold in a way that enables effective transfer learning. The sphere-based view provides insight into the rotational step of transfer learning using the Riemannian Procrustes analysis method and highlights the limitations of rotation. For effective transfer learning, selecting appropriate source data is essential for good performance. The pole ratio is shown to be an effective metric for selecting source data. The main contribution of the paper is the insight into the limitations of rotations on a Riemannian manifold; the usefulness of the pole ratio as a source selection metric is a natural extension of this insight. This paper focuses on Brain-Computer Interfaces (BCIs), but the sphere-based view of rotations of symmetric positive-definite matrix data and the pole ratio are applicable to any field that models two-class data using symmetric positive-definite matrices.

## 1 Introduction

**Figure 1:**
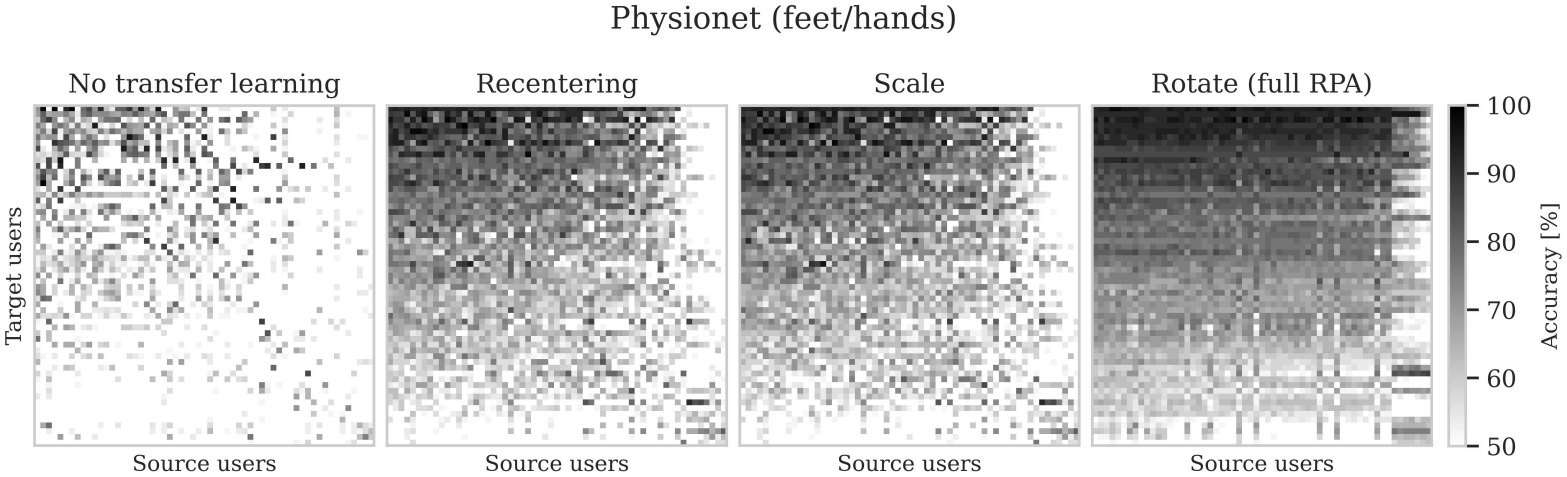
Transfer learning accuracies for all combinations of source and target users. The subfigure titles indicate the transfer learning step. Target users are in rows, and source users are in columns. Rows are sorted from top to bottom by descending row sum, and columns from left to right by descending column sum. Black indicates a transfer learning accuracy of 100%, whereas white indicates 50% or less. Despite full RPA, some source users are more beneficial than others, as seen from the different shades of gray within each row of the rightmost subplot. The band of source users to the right with deviant transfer learning in the rightmost subplot is further discussed in Section 5.2.

Transfer learning is often used in Brain-Computer Interfaces (BCIs) to reduce calibration time for a new user. Through transfer learning, data previously collected from another user (the source user) is used to train the BCI's underlying machine learning algorithms for the new user (the target user). The benefit of transfer learning is that less training data from the target user is needed, which enables a quicker startup of the BCI as the calibration time is reduced. The transfer learning performance is compared with the performance obtained when only the target data are used for training. There are many approaches to transfer learning for BCIs, where the aim often is to align the source data with the target data (Jayaram et al., 2018; Wu, 2025). This paper focuses on the Riemannian Procrustes Analysis (RPA) which is a state-of-the-art method for transfer learning in BCIs (Rodrigues et al., 2019b; Wu, 2025). It uses covariance matrices as features of the BCI data, which are symmetric positive-definite (SPD) matrices that lie on a Riemannian manifold of SPD matrices. The alignment of source to target data is done on this manifold in three steps: recentering, scaling, and rotation. The recentering step moves the source and target covariance matrices to a common reference point (typically the identity matrix). The scaling step scales the data so that the source and target data get comparable dispersions. These two steps operate without labels and constitute the unsupervised component of RPA. In contrast, the rotation step uses class labels and aims to rotate the source data to match the target data. After the source data has been aligned to target data with the RPA method, a classifier is trained on the source data and used to predict the class of the target data. In theory, any source user should benefit any target user, since the data is aligned by the RPA method. However, that is not always the case in practice (Rodrigues et al., 2019a), as shown in Figure 1. The rotation step is both computationally and labeling-wise more expensive than the first two steps and thus warrants further investigation. A better understanding of the RPA method's limitations serves as a first step toward improving transfer learning for BCIs and informs source data selection decisions.

Previous work has shown that transfer learning accuracy varies substantially across source-target user pairs and has identified the target user's intra-subject accuracy as a strong predictor of overall performance (Rodrigues et al., 2019a). In this paper, we study why some pairs of source-target users give higher transfer learning accuracy than others by analyzing the limitations of the RPA method's rotational step. We introduce a sphere-based view of rotations on the Riemannian manifold of SPD matrices, equipped with the affine-invariant Riemannian metric, to facilitate understanding. The key element of the sphere-based view is that the ray of scaled identity matrices is unaffected by rotation, thereby constraining it. Based on this sphere-based view, we propose the *pole ratio* metric, which indicates a point's position relative to this ray of unaffected points. The pole ratio is useful for characterizing a user's BCI data and thus informing decisions of source data selection for a new target user. The remainder of the paper develops the theoretical framework, introduces the pole ratio metric, and evaluates its effectiveness for source data selection on multiple BCI datasets.

## 2 Related works

The purpose of using transfer learning in BCIs is to reduce the calibration time for BCIs. By using data from a source user to train the underlying machine learning algorithms, less training data from the target user is required, resulting in shorter calibration time. Transfer learning is not a magic trick that solves all problems for BCIs; it has limitations. The limitations are method-specific, and in this paper, we study the RPA transfer learning method.

Transfer learning can be achieved either by aligning data or models between source and target domains (Jayaram et al., 2018). For BCIs, commonly used transfer learning approaches include domain adaptation, improved CSP algorithms, deep neural networks, and subspace learning (Wan et al., 2021; Ko et al., 2021). In this paper, the RPA transfer learning method presented in Rodrigues et al. (2019b) is studied. It is a subspace learning method where the data is aligned on a Riemannian manifold; see more details in Section 3.1 and Rodrigues et al. (2019b). Alternatives that are similar to the RPA algorithm include the Tangent space alignment (TSA) method (Bleuzé et al., 2022) and the Euclidean alignment (EA) method (Wu, 2025). The core of all these methods is to center the data, then rotate the source data to align with the target data. The RPA method does this on a Riemannian manifold, the TSA method does this in the tangent space to a Riemannian manifold, and the EA method does this in Euclidean space. We have chosen to study the RPA algorithm because BCI data, expressed as covariance matrices and analyzed on a Riemannian manifold of SPD matrices has been considered the state-of-the art method for motor imagery BCI data since its first introduction to the BCI community (Barachant et al., 2012). Gaining a deeper understanding of transfer learning on this manifold is essential for further development.

As presented in the introduction, the transfer learning performance depends on which source user is used for transfer learning. This motivates the need for source data selection methods. The arguably best approach is to evaluate performance with all available source users and select the best-performing (Wei et al., 2023). While this naive approach is optimal for small sets of source users, it is not viable for large sets, as evaluating transfer learning performance can be computationally expensive. However, it represents the best possible performance achievable and is referred to as the *Oracle* method in this paper. Resting-state EEG data can be compared between source and target users to select source data (Jeon et al., 2019). Iteratively including and excluding source data using a so-called sequential forward floating search, and evaluating performance with and without each subset of data, is another approach (Xu et al., 2021; Li et al., 2021). Selecting source data with class means as close as possible to the target data after recentering is an approach similar to the pole ratio approach presented in this paper (Orihara et al., 2023; Wu et al., 2023). The distance-based method is referred to as the *Distance* method in this paper.

Some transfer learning approaches use multiple source users, while others use only one. When multiple source users are used, the source data can be combined into a single dataset, or ensemble voting can be used to aggregate the classification predictions from each source user (Jayaram et al., 2018; Wan et al., 2021; Wu, 2025). In this paper, we present the pole ratio metric, which highlights the limitations of transfer learning on a Riemannian manifold. The pole ratio metric can be used to select source data; the question of how many source users to choose or how to combine them is out of scope for this paper.

## 3 Materials and methods

This section first presents selected aspects of Riemannian geometry to lay the foundation for the paper. Next, the mathematical details for the sphere-based view of the SPD data rotations and the pole ratio are presented. Then, methods for analyzing BCI data using the pole ratio are explained. Finally, the BCI datasets used in this paper are presented. The code for the paper can be found on github: https://gitlab.control.lth.se/FridaH/pole_ratio_public.

### 3.1 Riemannian geometry for BCI data

A thorough introduction to Riemannian geometry for BCI data is given in Yger et al. (2017). Here, a summary is given for context in this paper.

The covariance matrix $C_{i}=\frac{1}{s}X_{i}X_{i}^{⊤}$, is used as a feature for the EEG trial, $X_{i}\in {\mathbb{R}}^{n\times s}$ which has *n* channels and *s* samples. The label for the trial, i.e. the class for the EEG trial, is denoted *y*_*i*_∈{1, …, *K*} where *K* is the number of classes. *K* = 2 in this paper, meaning that each EEG trial comes from one of two classes as specified in Section 3.8. The covariance matrices lie on the Riemannian manifold of SPD matrices equipped with the Affine Invariant Riemannian Metric (AIRM), $P_{n}$, hereafter referred to as the *AIRM SPD manifold*. The AIRM gives the distance between two covariance matrices on this manifold $C_{i}\in P_{n}$ and $C_{j}\in P_{n}$, as


$$\begin{array}{l} \delta_{R}\left(C_{i},C_{j}\right)=||\log\left(C_{i}^{-1/2}C_{j}C_{i}^{-1/2}\right){||}_{F}, \end{array}$$ (1)

where log is the matrix logarithm. The above distance, Equation 1, is hereafter called the *Riemannian distance*.

The geometric mean, $M_{k}\in P_{n}$, for the covariance matrices $C_{i}\in P_{n}$ with label *y*_*i*_ = *k*, i.e. the geometric mean for the set $C_{k}=\left\{C_{i}\in P_{n}|y_{i}=k\right\}$, is defined as


$$\begin{array}{l} M_{k}=\text{arg}\underset{X\in P_{n}}{\min}\underset{C_{i}\in C_{k}}{\sum }\delta_{R}^{2}\left(X,C_{i}\right). \end{array}$$ (2)

In other words, the class mean is the covariance matrix that minimizes the squared distance to all covariance matrices with class label *k*.

Source and target data are indexed with superscripts S and T, respectively. For example, a covariance matrix for target data is denoted $C_{i}^{T}$ and the class mean for class *k* of the source data is denoted by $M_{k}^{S}$.

Transfer learning is done with the Riemannian Procrustes Analysis (RPA) transfer learning method (Rodrigues et al., 2019b), which is implemented in the open source Python package pyRiemann (Barachant et al., 2025). In the RPA method, the source and target data are first recentered to the identity matrix *I*, then scaled so the source and target data have the same dispersion, and finally, the source data is rotated so that the source class means $M_{k}^{S}$ more closely match the corresponding target class means $M_{k}^{T}$. Rotation of a matrix $C\in P_{n}$ is done with an orthogonal matrix *U*∈ℝ^*n*×*n*^, with *U*^⊤^*U* = *I*, via the congruence transform


$$\begin{array}{l} C_{rot}=U^{⊤}CU. \end{array}$$ (3)

The RPA algorithm obtians the rotation matrix *U* from an optmization problem on the manifold of orthogonal matrices where the sum of squared distances between $M_{k}^{S}$ and $M_{k}^{T}$ for all classes *k* is minimized. For more details on the RPA algorithm the interested reader is referred to the paper by Rodrigues et al. (2019b). After the source and target data have been aligned with the RPA method, classification is performed using the minimum distance to mean (MDM) classifier, using the Riemannian distance (Equation 1) and class means (Equation 2) as defined above.

### 3.2 Sphere-based view of SPD data rotation

The sphere-based view of SPD data rotation that is presented here highlights the limitations of the rotational step in the RPA method. The key element of this sphere-based view is that there is a ray of points that are unaffected by the rotation and points are bound to keep their intermediate distance under rotation. Thus, points cannot be rotated arbitrarily on the AIRM SPD manifold. The rotation can be compared to that of Earth, which has points on its rotational axis that are unaffected by the rotation. For example, the north and south poles lie on this ray of points unaffected by rotation. A point near the north pole before rotation will remain near the north pole after rotation. The ray of unaffected points on the AIRM SPD manifold is not a rotational axis as for Earth. Still, the comparison gives intuition for the rotational limitations on the AIRM SPD manifold. The following are the mathematical details of this sphere-based view of SPD data rotations on the AIRM SPD manifold.

As described by Rodrigues et al. (2019b), rotation is done with the congruence transform (Equation 3). Before rotation, the data is recentered to the identity matrix *I*, which serves as the rotation center. The identity is invariant under rotation since


$$\begin{array}{l} I_{rot}=U^{⊤}IU=I, \end{array}$$

i.e., the point *I* is unaffected by the rotation. Moreover, Rodrigues et al. (2019b) points out that as a result of the AIRM, all points preserve their Riemannian distances to each other under affine transformations, such as rotations, meaning


$$\begin{array}{l} \delta_{R}\left(C_{1},C_{2}\right)=\delta_{R}\left(U^{⊤}C_{1}U,U^{⊤}C_{2}U\right)=\delta_{R}\left(C_{1rot},C_{2rot}\right). \end{array}$$ (4)

Based on the above, the rotation of recentered data can be interpreted as a rotation around the identity matrix *I*. The novel element for the presented sphere-based view is that any scaled identity matrix λ*I* (an identity matrix *I* scaled with a scalar λ∈ℝ) is also invariant under rotation since


$$\begin{array}{l} {\left(\lambda I\right)}_{rot}=U^{⊤}\left(\lambda I\right)U=\lambda I. \end{array}$$ (5)

Thus, the ray of scaled identity matrices, {λ*I*:λ > 0}, consists of points unaffected by the rotation. We keep λ > 0 since we only consider SPD matrices.

We will now introduce what we call a *geodesic sphere*. The geodesic sphere is the sphere that is formed by all points $C\in P_{n}$ on the AIRM SPD manifold with distance *r* = δ_*R*_(*C, I*) to the identity matrix. We say that this geodesic sphere has a radius of *r*. The geodesic sphere intersects the ray {λ*I*:λ>0} in exactly two points, which we call the *north* and *south poles* of the sphere. The north and south poles are scaled identity matrices, λ*I*, with different values for λ. The north and south poles are unaffected by the rotation since they are part of the ray {λ*I*:λ>0}. The Riemannian distance (Equation 1) from a *n*×*n* scaled identity matrix λ*I* to the identity matrix is


$$\begin{array}{l} \delta_{R}\left(I,\lambda I\right)=\sqrt{n}|\log\lambda |. \end{array}$$

To find the λ for the north and south poles of a geodesic sphere with radius *r*, we solve


$$\begin{array}{l} \sqrt{n}|\log\lambda |=r \end{array}$$

for λ and get


$$\begin{array}{l} \lambda_{\pm }=\exp\left(\pm \frac{r}{\sqrt{n}}\right) \end{array}$$ (6)

with the north pole from the positive sign, λ_+_*I*, and the south pole from the negative sign, λ_−_*I*. From Equation 5 we have that the poles are invariant under rotation. That means that they act as fixed points under rotation on the geodesic sphere centered at *I* with radius *r*, similarly to the north and south poles on Earth.

When the points $C_{i}\in P_{n}$ are rotated on the manifold of SPD matrices, the rotated points keep all pairwise distances as defined in Equation 4. Specifically, they keep the distance to each other, to the identity matrix *r*_*i*_ = δ_*R*_(*I, C*_*i*_), to the north pole δ_*R*_(*C*_*i*_, λ_+_*I*), and to the south pole δ_*R*_(*C*_*i*_, λ_−_*I*) under rotation. Since the north and south poles, λ_+_*I* and λ_−_*I*, are fixed, and the points *C*_*i*_ keep their distance to them, the points *C*_*i*_ cannot be rotated arbitrarily; they are limited to staying at the same distance from the north and south poles as before rotation. The same is true for the distance to the identity matrix. This means that the points on the geodesic sphere stay on the sphere under rotation. The geodesic sphere can be rotated, but the north and south poles are fixed, which limits how the geodesic sphere can be rotated. This means that we can compare the rotation of an SPD matrix on the AIRM SPD manifold with that of a point on Earth. Rotation of the geodesic sphere is done in the high dimensional AIRM SPD manifold $P_{n}$ as opposed to Earth's rotation in three-dimensionall Euclidean space. The point is rotated along the surface of its sphere, keeping its distance to the fixed north and south poles. The ray {λ*I*:λ>0} is not a rotational axis, but points that are invariant under rotation.

### 3.3 Pole ratio

Rotation with the congurence transform Equation 3 on the AIRM SPD manifold keeps the north and south poles, λ_+_*I* respectively λ_−_*I*, fixed as described above. By analogy with how latitude describes position between the poles on Earth, we define a point's *pole ratio*, ρ, as its normalized position between the north and south poles on the geodesic sphere with radius *r*. The geodesic sphere, the north pole, and the south pole are defined above. For a point $C\in P_{n}$ on the geodesic sphere with radius *r* the pole ratio, ρ, is


$$\begin{array}{l} \rho =\frac{\delta_{R}\left(C,\lambda_{+}I\right)}{\delta_{R}\left(C,\lambda_{+}I\right)+\delta_{R}\left(C,\lambda_{-}I\right)}. \end{array}$$ (7)

A pole ratio of 0.5 means the point lies halfway between the poles, that is, on the equator if it were on Earth, while a pole ratio close to 0 means the point lies near the north pole and 1 means close to the south pole.

**Figure 2:**
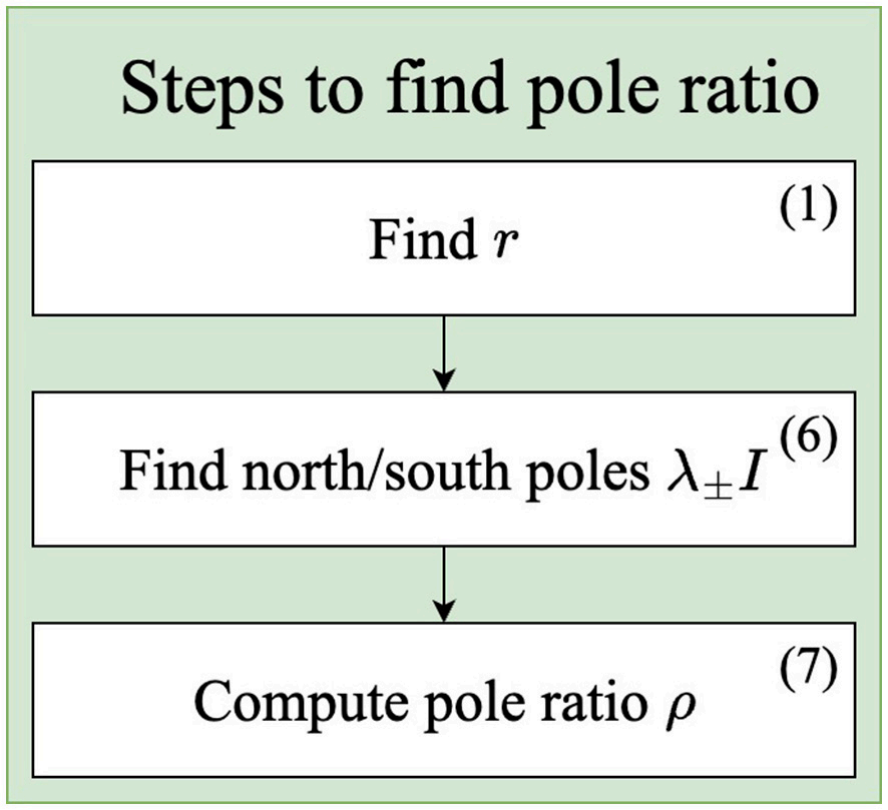
Diagram showing the steps to find the pole ratio for an SPD matrix. The numbers in parenthesis refer to the equation number used in the step.

To find the pole ratio for a point $C\in P_{n}$, the first step is to find the radius *r* for its geodesic sphere. In other words, the first step is to find the distance to the identity matrix *r* = δ_*R*_(*I, C*) with Equation 1. The next step is to find the north and south poles for the geodesic sphere with radius *r* from Equation 6. Finally, the pole ratio is found from Equation 7. The process of finding the pole ratio is summarized in Figure 2.

### 3.4 Direct visualization of SPD matrices

BCI covariance matrices lie on the typically high-dimensional AIRM SPD manifold $P_{n}$ and cannot be visualized directly. However, 2 × 2 SPD matrices on $P_{2}$ can be visualized by embedding each SPD matrix


$$\begin{array}{l} C=\left[\begin{array}{cc} c_{11} & c_{12}\\ c_{12} & c_{22} \end{array}\right] \end{array}$$

as the point (*c*_11_, *c*_12_, *c*_22_) in ℝ^3^.

### 3.5 Visualization of class means based on pole ratio

Given the sphere-based view and pole ratio metric introduced above, each class mean $M_{k}\in P_{n}$ for a user can be represented as a point on a circle. The radius for the circle, denoted *r*_*k*_, corresponds to the class mean's Riemannian distance to the identity matrix, *r*_*k*_ = δ_*R*_(*I, M*_*k*_). In other words, the radius for the circle is the same as the radius for the geodesic sphere that the class means lie on. The class means *M*_*k*_ are placed along this circle according to their pole ratios. The north pole is at the top of the circle and the south pole at the bottom. Thus, a point with a pole ratio close to 0 is at the top of the circle, 1 at the bottom, and 0.5 to the right or left.

Before computing class means from the data, the data is recentered to the identity matrix, *I*, and scaled to unit dispersion to ensure comparability across subjects. In other words, the first two steps of the RPA algorithm are performed before calculating the pole ratio.

### 3.6 Pole ratio for source data selection

The improvement in transfer learning accuracy relative to the intra-subject accuracy for a pair of source and target users is evaluated as


$$\begin{array}{l} \text{ΔAcc}=\text{Ac}{\text{c}}_{\text{TL}}-\text{Ac}{\text{c}}_{\text{intra}}^{T}\in \left[-100,100\right], \end{array}$$

where Acc_TL_ is the transfer learning accuracy for the pair of source-target users and $\text{Ac}{\text{c}}_{\text{intra}}^{T}$ is the intra-subject accuracy for the target user. If the ΔAcc>0, it means that the transfer learning accuracy is better than the intra-subject accuracy, and vice versa if ΔAcc < 0. If ΔAcc < 0, there is no benefit of doing transfer learning, and the selected source data is not good for the target user. If ΔAcc≈0, transfer learning remains beneficial, as one can use the source data instead of collecting additional target data, thereby reducing calibration time for the new target user.

For a pair of source and target users, the difference in pole ratio is calculated as


$$\begin{array}{l} \text{Δ}\rho =\rho^{S}-\rho^{T}\in \left[-1,1\right]. \end{array}$$

where ρ is the pole ratio and the superscript denotes source or target data. The absolute value of the difference in pole ratio, |Δρ|, is used for source data selection.

The statistical significance of the pole ratio as a metric for source data selection is assessed using a Welch's t-test. The hypothesis is that a pair of source and target users with a small difference in pole ratio, |Δρ|, yields significantly higher improvement in transfer learning accuracy, ΔAcc, than a pair of source and target users with a larger difference in pole ratio, |Δρ|. The cases in Table 1 are considered for analysis. Let 𝔼[ΔAcc(*x*)] be the expected ΔAcc value for the pairs of source and target users that have a |Δρ| which fulfills the requirement *x*. The cases in Table 1 compare inner regions *z* (with a smaller |Δρ|) with outer regions *q* (with larger |Δρ|) with the hypothesis test as


$$\begin{array}{ll} H_{0} & :𝔼\left[\text{ΔAcc}\left(z\right)\right]=𝔼\left[\text{ΔAcc}\left(q\right)\right]\\ H_{1} & :𝔼\left[\text{ΔAcc}\left(z\right)\right]>𝔼\left[\text{ΔAcc}\left(q\right)\right] \end{array}$$

The six cases compare inner regions with their closest outer regions.

**Table 1:** Requirements on pole ratio, |Δρ|, for each case for the inner regions *z* (small difference in |Δρ|) and outer regions *q* (large difference in |Δρ|).

Case	Inner region (*z*)	Outer region (*q*)
1	|Δρ| ≤ 0.2	0.2 < |Δρ| < ∞
2	|Δρ| ≤ 0.1	0.1 < |Δρ| < ∞
3	|Δρ| ≤ 0.1	0.1 < |Δρ| ≤ 0.2
4	|Δρ| ≤ 0.05	0.05 < |Δρ| < ∞
5	|Δρ| ≤ 0.05	0.05 < |Δρ| ≤ 0.2
6	|Δρ| ≤ 0.05	0.05 < |Δρ| ≤ 0.1

The regions in each case are compared using Welch's t-tests.

### 3.7 Comparison of source data selection methods

We compare the transfer learning performance for the source selection methods listed below. The methods are representative of source selection mehtods that select source data independent of the transfer learning and classification as opposed to methods where all source data is used. For a target user in a dataset, each source selection method selects a source user. The transfer learning performance for the selected source user is evaluated and compared with the other methods' selections. The source selection process is repeated for all users in the dataset, so each user acts as the target user once.

- Random: The source user is selected at random.
- Intra-subject: The performance if no transfer learning is performed. It serves as a baseline for the performance that would be achieved if only the target data were used for training.
- Distance: The source user with class means closest to the target user after the recentering and scaling step in the RPA transfer learning algorithm. This method is similar to what they did in (Orihara et al., 2023).
- Source intra-subject: The source user is selected as the one with the highest intra-subject performance.
- Pole ratio: Source data is selected as the one with the smallest difference in pole ratio to the target user.
- Oracle: The performance with the source user who yields the highest transfer learning performance. It represents the best possible choice of source data. The method is similar to that used in (Wei et al., 2023).

### 3.8 BCI data

The public EEG datasets summarized in Table 2 are used in this paper. The datasets include labeled EEG data for motor imagery (MI) tasks and are accessed through the open source MOABB project (Aristimunha et al., 2025; Jayaram and Barachant, 2018). The ten EEG channels FC3, FC4, C5, C3, C1, C2, C4, C6, CP3, and CP4 are used because they cover the motor cortex (Graimann et al., 2010). The EEG data is bandpass filtered in the range 7-35 Hz as it covers the frequency bands where interesting EEG features are expected to appear (Pfurtscheller and Neuper, 2010). Data from each trial from 1 sec after the cue onset until 2 sec after the cue onset are used to avoid any initial transients from the stimulus onset. The 10 × 10 covariance matrix $C_{i}=\frac{1}{s}X_{i}X_{i}^{⊤}$ is used to represent trial *i*'s data, which is calculated from the EEG data $X_{i}\in {\mathbb{R}}^{10\times s}$, which has ten channels and *s* xsamples.

**Table 2:** Information about datasets.

Dataset	Classes (orange vs. blue)	Nbr subjects	Trials per class	Reference
BCI-IV 2a (l/r)	left vs. right hand	9	144	Tangermann et al., 2012
BCI-IV 2a (f/t)	feet vs. tongue	9	144	Tangermann et al., 2012
Cho	left vs. right hand	52	100	Cho et al., 2017
Dreyer	left vs. right hand	87	120	Benaroch et al., 2022; Pillette et al., 2021
Lee	left vs. right hand	54	100	Lee et al., 2019
Physionet (f/h)	feet vs. hands	109	23	Goldberger et al., 2000; Schalk et al., 2004
Physionet (l/r)	left vs. right hand	109	23	Goldberger et al., 2000; Schalk et al., 2004

The orange vs. blue class refer to the color of the classes in Figure 4 and Table 3.

### 3.9 Data split for classification evaluation

Classification is done with the minimum distance to mean (MDM) classifier using the Riemannian distance (Equation 1). For intra-subject accuracy, 75% of the available data is used as training data to train the MDM classifier, with the remaining 25% used as test data to evaluate classification accuracy. Four repetitions with different data splits are used to compute the average intra-subject classification accuracy.

For transfer learning accuracy, 75% of the available target data is used as target training data for the RPA algorithm (Rodrigues et al., 2019b), and the remaining 25% is used as test data to evaluate classification accuracy. The MDM classifier is trained on all available source data (after it has been transformed by the RPA algorithm) and on no target data. The target training data is thus used only for transfer learning with the RPA method. Four repetitions with different target data splits are used to compute the average transfer learning classification accuracy.

## 4 Results

This section presents the results. The results are discussed in the following Discussion section, Section 5. First, the visualization of 2 × 2 SPD matrices is presented as an example of the sphere-based view to illustrate the geodesic sphere and the rotation of SPD matrices. Then, the pole ratio for different datasets is presented, along with an illustration of class means based on the pole ratio. Finally, the results of when the pole ratio is used for source data selection are presented.

### 4.1 [

Sphere-based view of 2 x 2 SPD rotations]Sphere-based view of 2 × 2 SPD rotations

**Figure 3:**
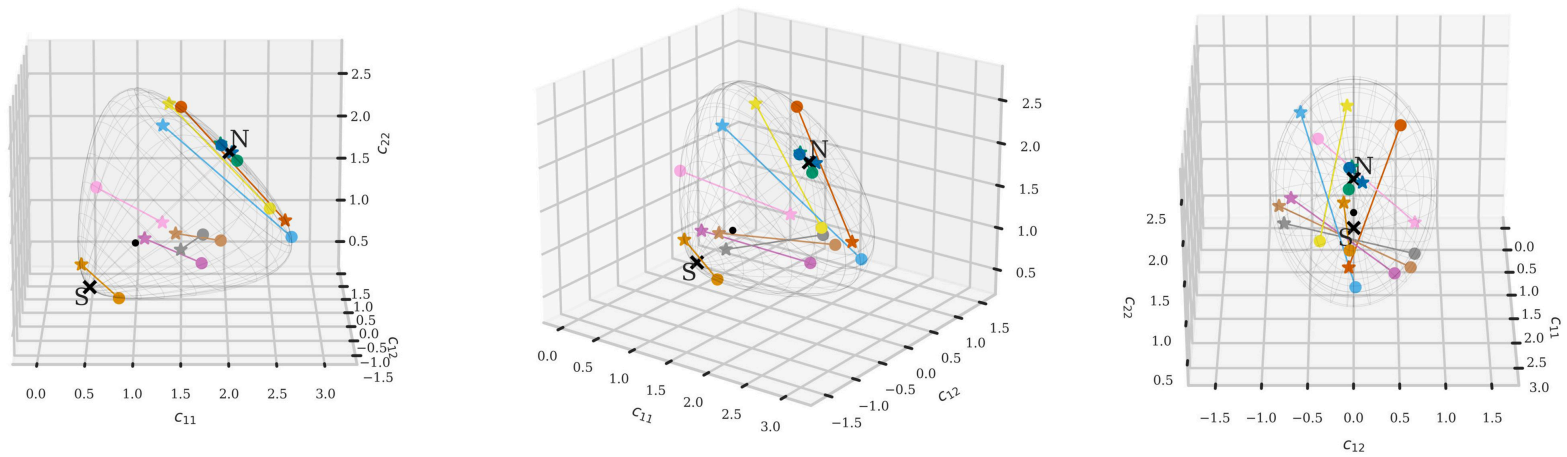
Visualization of 2 × 2 SPD matrices and their rotation from different angles. The surface shows the geodesic sphere with Riemannian distance *r* = δ_*R*_(*C, I*) = 1. The north pole is marked with a cross and an N, the south pole is marked with a cross and an S. The star-shaped points are points before rotation, and the dot-shaped points are the points after rotation. The lines connect the dots for visual purposes. The black point is the identity matrix and does not lie on the geodesic sphere; all other points lie on it.

Figure 3 shows the geodesic sphere defined by *r* = δ_*R*_(*C, I*) = 1 for 2 × 2 SPD matrices, viewed from three different angles. The north pole is marked with a cross and an N, the south pole is marked with a cross and an S. On the geodesic sphere, a few points are marked. The points are rotated with an orthonormal matrix *U* with Equation 3. A star indicates the starting point and a dot the endpoint for the rotation; the line segments connect the starting and endpoints for visual aid. The identity matrix is the black dot. After rotation, the points preserve their distances from the identity matrix, their pairwise distances, and their pole ratio, thus, remain on the geodesic sphere.

All distances are with the Riemannian distance Equation 1 on the AIRM SPD manifold. Since the Riemannian distance is used to measure distances, the geodesic sphere shown in Figure 3 is not ball-shaped as one would expect for a sphere if the Euclidean metric is used. However, measured with the Riemannian distance, the surface shown in Figure 3 is a sphere with every point on the surface at a distance of one to the identity matrix, exactly as one intuitively expects from a sphere.

### 4.2 Pole ratio for BCI data

**Figure 4:**
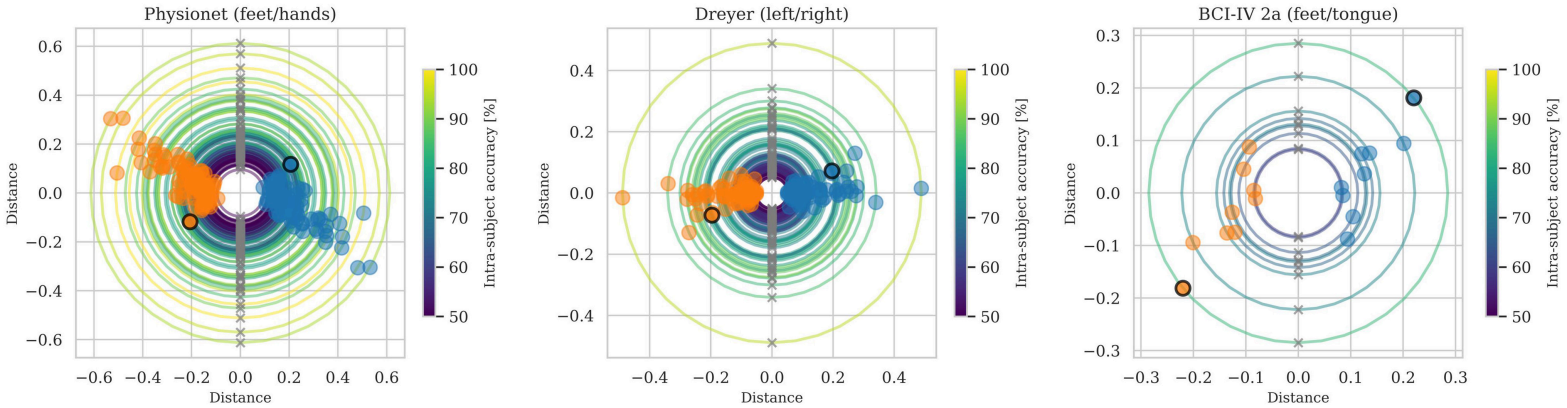
Class means visualized based on pole ratio for each user in three datasets. The radius of each circle corresponds to the class means' distance to the identity matrix, i.e., the radius of the geodesic sphere the class means lie on. The north and south poles of the geodesic sphere are marked with an x at the top and bottom of each circle, respectively. Blue and orange dots represent the class means, placed along the circle according to the class means' pole ratio. Class means close to the top of the circle has a pole ratio close to 0, and class means close to the bottom of the circle has a pole ratio close to 1. A class mean halfway between the top and bottom (to the right or left of the circle) has a pole ratio close to 0.5. The distances on the x- and y-axes are the Riemannian distance from the identity matrix.

Figure 4 visualizes the class means' positions based on pole ratio, as described in Section 3.5, for all users in three datasets, as indicated by the subplots' titles. Each user's data is represented by a circle, a blue dot, and an orange dot. The color of the circle represents the intra-subject accuracy, i.e. the accuracy if only data from the target user is used for training. Circles with larger radii generally correspond to higher intra-subject accuracy, as indicated by the colors. The north and south poles of the geodesic sphere are visualized with crosses in Figure 4 at the top and bottom of the circle, respectively. The class means, the blue and orange dots, are placed along the circle according to the pole ratio. If the pole ratio is close to 0, the class mean is close to the north pole, and if the pole ratio is close to 1, the class mean is close to the south pole. The data from all users in a dataset is plotted in the same subfigure; thus, the subfigures show multiple circles, blue and orange dots, and crosses. In each subplot, data from a single subject is highlighted by a black outline around the blue and orange data points.

In this paper, two-class datasets are used, meaning that the identity matrix is placed approximately between the class means after recentering. As a result of this, both classes are at approximately the same distance from the identity matrix, and the pole ratio for the classes satisfies ρ_*o*_+ρ_*b*_ = 1 with the index indicating the orange or blue class. This is visually seen in Figure 4, where the blue and orange dots are on opposite sides of the circle for a given subject. For unbalanced datasets (unequal sample counts per class) or datasets with more than two classes, the class means may lie at different distances from the identity matrix. In such a case, the class means for a user in Figure 4 would not lie on the same circle and would not be opposite each other, as in the results here.

The average pole ratio and its standard deviation for the blue class, as defined in Table 2, for each dataset across subjects are presented in Table 3. The standard deviation is approximately the same across all datasets except BCI-IV 2a (f/t), which has a higher standard deviation.

**Table 3:** Mean pole ratio and its standard deviation for the blue class as defined in Table 2 across subjects for each dataset.

Dataset	Pole ratio	
	Mean	Std
BCI-IV 2a (l/r)	0.44	±0.06
BCI-IV 2a (f/t)	0.45	±0.14
Cho	0.50	±0.06
Dreyer	0.49	±0.07
Lee	0.46	±0.07
Physionet (f/h)	0.55	±0.09
Physionet (l/r)	0.49	±0.07

### 4.3 Pole ratio for source data selection

**Figure 5:**
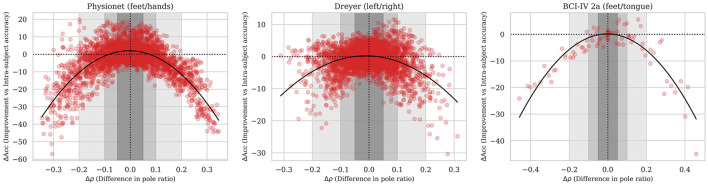
ΔAcc as a function of Δρ for three datasets for all pairs of source and target users in the datasets. The gray regions are visual aids for the statistical tests in Table 1 with results presented in Table 4.

Figure 5 shows the ΔAcc as a function of Δρ for three datasets. Each red dot corresponds to a pair of source and target users. All pairs of source and target users for each dataset are plotted. The black line is a fitted second-order polynomial trend line highlighting a clear trend with a smaller |Δρ| giving a higher ΔAcc. The observed negative values for low |Δρ| are further discussed in Section 5.4. The shaded areas in the subfigures are connected to the colorboxes in Table 4.

**Table 4:** Statistical results for hypothesis tests in Table 1.

	Cases					
	1	2	3	4	5	6
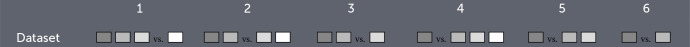						
BCI-IV 2a (l/r)	– (ns)	2 (*)	– (ns)	2 (**)	2 (**)	1 (*)
BCI-IV 2a (f/t)	15 (**)	10 (**)	3 (**)	8 (**)	2 (**)	– (ns)
Cho	6 (**)	3 (**)	2 (**)	2 (**)	2 (**)	1 (**)
Dreyer	6 (**)	3 (**)	3 (**)	2 (**)	2 (**)	1 (**)
Lee	9 (*)	3 (**)	3 (**)	2 (**)	2 (**)	1 (*)
Physionet (f/h)	19 (**)	11 (**)	6 (**)	7 (**)	3 (**)	1 (**)
Physionet (l/r)	14 (**)	8 (**)	6 (**)	5 (**)	4 (**)	1 (**)
Average	11	6	4	4	2	1

Values show the difference in expected value for ΔAcc between inner (*z*) and outer (*q*) regions, rounded to the closest integer. Significance is indicated in parentheses: (*) *p* < 0.05, (**) *p* < 0.01, and ns means non-significance. The color boxes are a visual aid for comparision with the highlighted regions in Figure 5.

Table 4 shows the average ΔAcc across subjects and results for the statistical test for all cases for all datasets. The average ΔAcc for each case across datasets is reported at the end of the table. The BCI-IV 2a datasets are the only ones with cases that are not statistically significant. These datasets have fewer subjects than the other datasets. For the other datasets, statistical significance is observed in all cases (*p* < 0.05), with most cases meeting the stricter *p* < 0.01 threshold.

**Figure 6:**
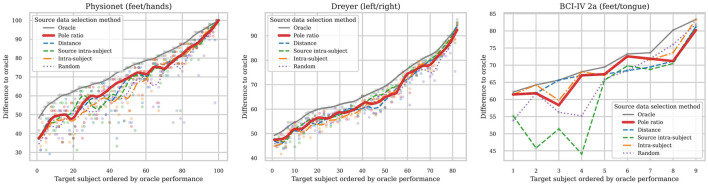
Comparison of source data selection methods for three datasets. On the x-axis, the subjects are ordered from left to right in increasing transfer learning performance with the Oracle method. The y-axis is the transfer learning performance. The dots show the raw data, and the lines are a smoothed trendline of this data.

Figure 6 shows the comparision of source data selection methods. The subjects are ordered on the x-axis from left to right in increasing transfer learning performance with the Oracle method. The y-axis is the transfer learning performance. The dots show the raw data, and the lines are smooth trendlines of this data. The oracle method is the best. The pole ratio method is generally better than the random and intra-subject methods and has similar performance to the other methods. These results and the source data selection methods are further discussed in Section 5.4.

## 5 Discussion

This section first discusses the presented sphere-based view of SPD data rotations and the pole ratio. Then the discussion turns to the results from BCI data. Finally, several promising directions for future research are discussed. The main contribution of this paper is the intuition for the limitations of transfer learning with the RPA method, given by the sphere-based view of SPD data rotations and the pole ratio. The applicability of the pole ratio as a source selection method is a natural consequence of this. However, the paper does not claim that the pole ratio is the best metric for source data selection; considering the pole ratio is important, but it should be combined with other metrics for optimal performance.

### 5.1 Sphere-based view of SPD data rotation

To briefly recap the sphere-based view: The first cornerstone of the sphere-based view is that all rotated points' distances from the identity matrix and each other are preserved under rotation. The second cornerstone is that points on the ray {λ*I*:λ>0} are unaffected by the rotation. These two cornerstorns constrains how points on the AIRM SPD manifold can be moved by rotation. Under rotation, a point moves along the surface of the geodesic sphere, keeping its distance to the geodesic sphere's north and south poles, which are invariant under rotation. The rotation of SPD matrices is analogous to Earth's rotation, with points moving along the surface of the sphere and a ray of points unaffected by it. If the rotation was not constrained by this ray of unaffected points, the points could be moved arbitrarily around the geodesic sphere. However, that is not the case.

The geodesic sphere for 2 × 2 SPD matrices shown in Figure 3 illustrates the key elements of the sphere-based view of SPD data rotations. Specifically, that the points keep their relative distance to all other points under rotation, and the ray {λ*I*:λ>0} is unaffected by rotation. The covariance matrices from EEG data are typically larger than 2 × 2. In this paper, they are 10 × 10. Such large covariance matrices cannot be visualized in three dimensions. However, the ray {λ*I*:λ>0} is still unaffected by the rotation, and the rotation still preserves the points' relative distances, which limits how the points can be moved. So the intuition from the geodesic sphere in the 2 × 2 case remains applicable and provides insight into the limitations of SPD rotation for matrices of any size.

Rotation in the RPA method is done on all source data with the orthogonal matrix *U* as described by Equation 3. The RPA method finds the matrix *U* that rotates the source data so that the distance between the class means $M_{k}^{S}$ and $M_{k}^{T}$ is minimized. The rotation with *U* is then applied to all source data, $C_{i}^{S}$, not just the class means. That means that all points, $C_{i}^{S}$, in the source data are rotated simultaneously with the same *U*. Thus, all points, $C_{i}^{S}$, maintain their distance from the identity matrix and each other. The target data is not rotated. Since the target data is fixed and the source data is rotated, the distance between the source and target data can be reduced, meaning that the source data aligns better with the target data.

The sphere-based view of SPD data rotations is valid and informative for rotations on the AIRM SPD manifold. If the SPD data is projected to the tangent space of the AIRM SPD manifold and transformed there, such as in the tangent space alignment method (Bleuzé et al., 2022), the sphere-based view does not give intuition to the transformations. The sphere-based view and pole ratio can still provide intuition for how the data is placed on the AIRM SPD manifold before projection to the tangent space, but not for how the data behaves under transformations in the tangent space. With further research, the pole ratio and the sphere-based view could likely be translated into the tangent space of the AIRM SPD manifold.

### 5.2 Pole ratio to describe limitations of transfer learning

For the source data to align with the target data, the two datasets must be placed on the AIRM SPD manifold so that the source data can be rotated to match the target data. The rotation is as discussed above, limited by the ray {λ*I*:λ>0} of unaffected points. Since a point keeps its distance to the north pole during rotation, the source and target data must have the same relative position to the ray of unaffected points for the rotation to be able to align the data. This is exactly what the pole ratio describes: a point's position relative to the north and south poles on the geodesic sphere. If the pole ratio is the same, the source data's class means can be rotated to the same position as the target data; if the pole ratio is different, the source data's class means can be rotated to be closer to the target data, but will never be in the same position. Looking at Figure 4, it means that users with class means in the same direction will be beneficial for transfer learning, while users with class means in different directions won't.

In Figure 1 there is a band of source users to the right that gives a deviant transfer learning performance than the rest of the source users. Looking at the left subplot in Figure 4, the highlighted data (black outline) is a bit off compared to the majority of the data. This highlighted user and the others in the same direction are those that appear in the band of source users with deviant transfer learning performance.

### 5.3 Pole ratio for BCI data

The average pole ratios for the datasets across subjects are close to 0.5 as reported in Table 3, indicating that the data generally lies halfway between the north and south poles. The BCI-IV 2a (f/t) dataset shows a higher standard deviation for the pole ratio than the other datasets. This could indicate that the MI task of imagining moving the feet and tongue is more diverse among users than, for example, the MI task of imagining moving the right or left hand. Based on the knowledge of how brain activity during MI tasks generates EEG data, theoretically expected values for the pole ratio could be found. For example, the north and south poles correspond to covariance matrices where all off-diagonal elements are zero and the on-diagonal elements are the same, meaning that there is no cross-correlation between the electrodes. For EEG data, there are expected to be a lot of cross-correlations, so a class mean placed very close to the north or south pole would be unexpected. If the expected pole ratio for an MI task were known, it could serve as a metric for assessing how well the user performed the task. If the pole ratio differed significantly from the expected value, another MI task might be more suitable. However, further research is needed to make any claims about theoretically expected pole ratios.

The observed trend in Figure 4 that a smaller radius correlates with lower intra-subject accuracy, as indicated by the circle's color, is consistent with intuition. If the data from the two classes are well-separated, their means will be far apart, resulting in a larger radius of the circle. Well-separated data typically yields a high classification accuracy. On the contrary, if the data from the two classes overlap a lot, the class means will be placed close to each other, resulting in a smaller radius of the circle, and the classification accuracy will be lower as a result of the misclassification of the overlapping data.

### 5.4 Pole ratio for source data selection for transfer learning

To use the pole ratio for source data selection, the first step is to collect training data from the target user to compute the target user's pole ratio. Then, source users with a pole ratio similar to that of the target users are evaluated for transfer learning. The source data is available offline, and the pole ratio for each source user can be computed in advance. The pole ratio for the target data must, however, be calculated online. The pole ratio is computed from the class means *M*_*k*_, which requires sufficient user training data to yield a robust estimate. How much training data is needed from the target user for such an estimate is outside the scope of this paper. The pole ratio describes where the class means of the data are placed on the geodesic sphere, but contains no information on how the data is distributed around these class means. An addition to the pole ratio, which describes the full data and not only the class means, is potentially beneficial and an interesting path for future research.

The statistical analysis of the results shows that selecting a source user with a pole ratio similar to that of the target user is beneficial for transfer learning. Thus, the pole ratio can serve as a metric for selecting source data. Looking at case six in Table 4, the average ΔAcc across datasets is one. Even though the result is statistically significant, one could argue that the gain is small. Still, if one were to choose a source user for a target user based solely on the pole ratio, the best choice would be the source user with the pole ratio most similar to the target user's. The trend lines that are shown in Figure 5 are fitted to each dataset individually. They show that there is a trend correlating a smaller |Δρ| to a higher ΔAcc. As seen from the distribution of the data in the figure, one cannot leave any guarantees such that if |Δρ| is smaller than *x*, ΔAcc will be larger than *y*. However, based on the statistical results, one can say that selecting a source user with a smaller |Δρ| for the target user will give a higher expected value for ΔAcc than if a source user with a higher |Δρ| for the target user is used. So basically, selecting a source user with a pole ratio similar to the target user's reduces the risk of selecting a poor source user, but does not guarantee selecting the optimal source user. Target and source users with similar pole ratios have no theoretical limitations on transfer learning performance, but the data distributions themselves will affect it. Thus, the pole ratio should be combined with other metrics for optimal source data selection.

The ΔAcc plotted in Figure 5 is adjusted for the intra-subject accuracy to give a fair comparison across subjects. The intra-subject accuracy is found from 75% of the target training data as described in Section 3.9. Thus, the intra-subject represents fairly good performance for the target user, as a large amount of training data is used. In a real BCI calibration setting, the amount of training data is limited, and the intra-subject accuracy would be lower. Thus, even some of the source users with negative ΔAcc would be beneficial, but still, the higher the better, of course. To further filter out the optimal source data, the pole ratio would need to be combined with other metrics. Since the aim of this paper is to provide insight into the limitations of transfer learning with the RPA method, combining the pole ratio with other metrics for refined source data selection is left for future work.

When comparing the source data selection methods in Figure 6 the Oracle method outperforms the others, as expected. The random method performs relatively well, particularly on the Dreyer dataset. This is somewhat expected since the difference between the source users for the Dreyer data set is relatively small, as seen in Figure 5. So by not selecting a bad source user, the performance is fairly good. Looking at the Dreyer dataset in Figure 4, we can see that most users are in the same direction, i.e. have a similar pole ratio, this once again strengthens the argument that the pole ratio can be used to not select a bad source user, but to select the best, the pole ratio needs to be combined with other metrics describing the data. For the Physionet dataset, the difference between users is larger, and the Random method performs worse than the other methods. In other words, if there are more differences in the source and target data, the more important it is to make informed source data selections. As seen for the Physionet dataset in Figure 6, using the pole ratio metric for source data selection is comparable to, and sometimes better than, other methods. But as previously pointed out: the aim of this paper is not to present the pole ratio as the best source data selection method, but to provide insight into and understanding of the rotation of SPD matrices on the AIRM SPD manifold. Based on this understanding, selecting source data with a pole ratio similar to that of the target data is important, but not the only factor to consider.

### 5.5 Future work

Finding a theoretical value of the pole ratio, based on how brain signals are generated and measured with EEG, is an interesting topic for future studies. What pole ratio values to expect require more research; what we do know is that a pole ratio of 0 or 1 is highly unlikely, as discussed previously.

This paper considers datasets with two classes. For more than two classes, there are more degrees of freedom in how the class means are placed on the AIRM SPD manifold around the identity matrix after recentering. This implies that the class means are not placed opposite on the geodesic sphere, as in the two-class case. Thus, the pole ratio for all classes, as well as some metric to describe the class means' position relative to each other on the geodesic sphere, would be needed to predict how well the source and target data can be aligned after transfer learning. Exactly how to describe the class mean's position on the geodesic sphere in the multi-class case is an alluring path for future research.

The pole ratio, as a metric for source data selection, is based on the observation that the so-called north and south poles remain unchanged under rotation. A fair question to ask based on this is “Why can't we do rotation in another way so there is no ray {λ*I*:λ>0} of unaffected points?” If one were to find another transformation that can rotate the points arbitrarily around the identity matrix while still working on the AIRM SPD manifold, transfer learning for BCIs would take a huge leap forward. Until then, the presented sphere-based view of the goedesic sphere provides important insights into the limitations of SPD data rotation on the AIRM SPD manifold, and the novel pole ratio remains a strong metric for characterizing which data are suitable for transfer learning. How to combine the pole ratio metric with other metrics to find the optimal source data is a topic for future research. A parallel direction for future work is to study whether the pole ratio metric provides any information about the performance with other transfer learning methods, such as the TSA method.

## 6 Conclusion

This paper studies the limitations of transfer learning on a Riemannian manifold of SPD matrices with the RPA transfer learning method. The paper introduces a sphere-based view of SPD data rotations, providing intuition into the limitations of transfer learning with the RPA method. The key element is that the rotation is not free; the ray of scaled identity matrices, {λ*I*:λ>0}, is unaffected by it. The presented pole ratio metric describes a data point's position on the so-called geodesic sphere relative to the sphere's invariant points, called the north and south poles. The pole ratio is a relevant metric for source data selection in transfer learning, as it indicates how similar two users are and how well their data can be aligned by the RPA transfer learning method. Multiple publicly available BCI datasets are used to analyze the pole ratio and its applicability for source data selection for transfer learning.

This paper has two main contributions: a sphere-based view of SPD rotations that provides intuition into their limitations, and the pole ratio, which quantifies a data point's orientation within this sphere-based view. The pole ratio is shown to be a useful metric for selecting source data.

## Data Availability

Publicly available datasets were analyzed in this study. This data can be found here: https://moabb.neurotechx.com/docs/index.html.
